# Multiparametric Magnetic Resonance Imaging in a Prospective Cohort of Living Kidney Donors, Recipients, and Healthy Controls and Correlations With Markers of Renal Function, Fibrosis, and Aging (MPRENAL): Protocol for a Longitudinal Observational Study

**DOI:** 10.2196/97152

**Published:** 2026-10-28

**Authors:** Patrick Schjelderup, Steffen Ringgaard, Jens Brøndum Frøkjær, Jens Dam Jensen, Søren Krag, Jens Randel Nyengaard, Christoffer Laustsen, Niels Henrik Buus, Lars Jødal, Karin Skov, My Hanna Sofia Svensson

**Affiliations:** 1 Department of Nephrology, Aalborg University Hospital, Mølleparkvej 4, Aalborg, Denmark, +45 97663700; 2 The MR Research Centre, Department of Clinical Medicine, Aarhus University, Aarhus, Denmark; 3 Department of Radiology, Aalborg University Hospital, Aalborg, Denmark; 4 Department of Clinical Medicine, Aalborg University, Aalborg, Denmark; 5 Department of Internal Medicine, Aalborg University Hospital, Hobro, Denmark; 6 Department of Pathology, Aarhus University Hospital, Aarhus, Denmark; 7 Core Center for Molecular Morphology, Department of Clinical Medicine, Aarhus University, Aarhus, Denmark; 8 Department of Renal Medicine, Aarhus University Hospital, Aarhus, Denmark; 9 Department of Nuclear Medicine, Aalborg University Hospital, Aalborg, Denmark

**Keywords:** multiparametric magnetic resonance imaging, kidney transplantation, living donors, biomarker, fibrosis, chronic kidney disease, glomerular filtration rate, protocol

## Abstract

**Background:**

Renal fibrosis is the final common pathway of chronic kidney disease progression and a critical histological predictor of renal function decline and allograft failure. Routine clinical monitoring with estimated glomerular filtration rate (eGFR) and albuminuria is insensitive to fibrosis development. Renal biopsy is invasive and subject to sampling bias, which limits repeated assessment, thus necessitating noninvasive imaging biomarkers. Multiparametric magnetic resonance imaging (mpMRI) characterizes renal structure and function noninvasively. Individual MRI measures have been associated with fibrosis and with declining renal function. However, longitudinal validation against histology and measured glomerular filtration rate (mGFR) remains limited.

**Objective:**

This prospective longitudinal observational study aims to validate mpMRI against histological fibrosis assessed by kidney biopsy in kidney transplant recipients (KTRs), and compare the time-dependent trajectories of imaging and functional markers across living kidney donors (LKDs), KTRs, and healthy control (HC) cohorts.

**Methods:**

The planned sample includes 32 LKDs, 32 KTRs, and 32 HCs. Up to 40 KTRs may be recruited to account for attrition. Baseline assessments are performed before transplantation for LKDs and KTRs, and at enrollment for HCs, with follow-up assessments at 3, 12, and 24 months. mpMRI is performed at 3 tesla (3 T). The MRI protocol includes structural longitudinal relaxation time (T1)–weighted and T2-weighted imaging, T1 mapping, T2 mapping, transverse relaxation time (T2*) mapping, diffusion-weighted imaging, pseudocontinuous arterial spin labeling, non–contrast enhanced angiography, and phase-contrast measurement of renal artery flow. Glomerular filtration rate will be measured by [^99m^Tc]Tc-diethylenetriaminepentaacetic acid (DTPA) clearance. A baseline allograft biopsy is taken during transplantation surgery, with protocol biopsies at 3, 12, and 24 months. Fibrosis is quantified using stereology. The primary outcome is the longitudinal association between the corticomedullary T1 difference and histologically determined renal fibrosis in KTRs across all 4 biopsy time points. Secondary analyses include longitudinal divergence of MRI and functional markers across cohorts, diagnostic performance for fibrosis, and predictive value for mGFR decline in KTRs and LKDs. Linear mixed models will be used to study longitudinal associations. Three additional MRI measures will be tested in a fixed sequence to control multiplicity. Receiver operating characteristic curve analysis will assess diagnostic performance.

**Results:**

The study was funded in October 2023. Recruitment for the multiparametric renal magnetic resonance imaging (MPRENAL) study commenced on October 29, 2024. As of March 2026, 42 participants were enrolled, and data collection is ongoing. Recruitment is anticipated to close in February 2027, with final study visits by March 2029. The primary results are expected to be published in 2030.

**Conclusions:**

Successful completion of this study is expected to provide robust histological validation of mpMRI and establish its utility as a noninvasive tool for monitoring renal health.

## Introduction

### Clinical Importance of Renal Fibrosis

Renal fibrosis is the final common pathway of chronic kidney disease progression and a critical histological predictor of renal function decline and allograft failure [1-4]. In kidney transplantation, progressive tubulointerstitial fibrosis contributes to long-term graft dysfunction and may develop before substantial changes in routinely measured kidney function are detected. In parallel, living kidney donors (LKDs) provide a complementary model of renal adaptation after nephron mass reduction. Long-term renal risk after donation is influenced by pre-donation kidney function, age, and structural kidney characteristics such as cortical volume [5-7]. Including kidney transplant recipients (KTRs), LKDs, and healthy control (HC) cohorts enables comparison of imaging and functional trajectories across transplanted, solitary, and healthy aging kidneys.

### Limitations of Current Monitoring Tools

Routine clinical monitoring with estimated glomerular filtration rate (eGFR) and albuminuria is insensitive to the development of fibrosis [8]. Measured GFR (mGFR) offers a more accurate assessment of kidney function than eGFR, particularly in settings where creatinine-based estimates may be unreliable, but mGFR does not directly quantify tissue remodeling. Renal biopsy remains the reference standard for histological assessment of fibrosis; however, its invasive nature limits repeated assessment. It is also affected by sampling variability because only a small fraction of the kidney is examined [9,10]. These limitations create a need for noninvasive biomarkers capable of capturing structural, functional, and molecular changes over time.

### Multiparametric Magnetic Resonance Imaging as a Noninvasive Biomarker Platform

Multiparametric magnetic resonance imaging (mpMRI), defined as the combination of several MRI measures sensitive to different biophysical tissue properties in a single scan session, offers noninvasive whole-organ characterization of renal structure and function. MRI measures such as longitudinal relaxation time (T1) mapping, diffusion-weighted imaging (DWI) with apparent diffusion coefficient (ADC), arterial spin labeling (ASL), and blood oxygen level-dependent imaging (BOLD) may provide complementary information on tissue water content, apparent water diffusion, perfusion, and oxygenation. ADC is derived from DWI and quantifies the apparent mobility of water molecules; changes in ADC may therefore indirectly reflect alterations in tissue microstructure, including tubular and interstitial architecture, cellularity, edema, and fibrosis. Previous work has indicated that T1 mapping, diffusion-based measures, and ASL-derived perfusion are associated with renal fibrosis, whereas T1 mapping, diffusion measures, and BOLD have also been linked to declining renal function [11-14]. Figure 1 illustrates the conceptual link between selected MRI measures and fibrosis development.

A major challenge in renal mpMRI research is the lack of standardized protocols for image acquisition, postprocessing, and analysis. This limits comparability across studies and centers. In accordance with recommendations from PARENCHIMA, the present protocol provides detailed information on study design, patient selection, MRI acquisition, postprocessing, and image analysis [15].

**Figure 1.**
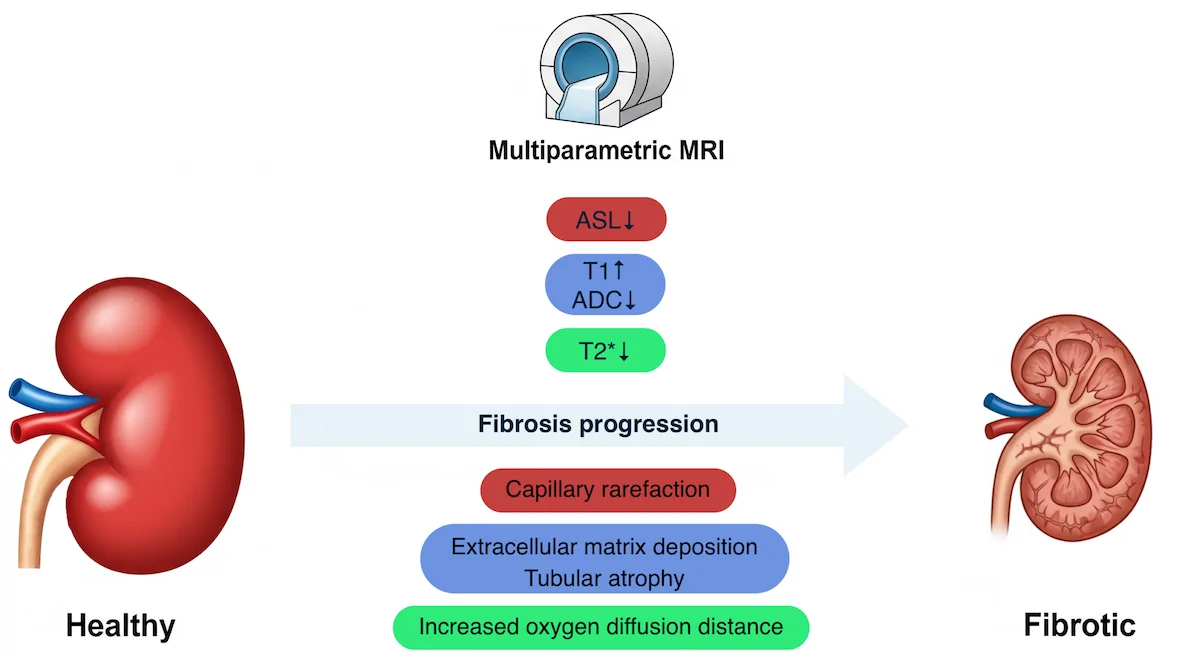
Conceptual link between renal fibrosis and multiparametric magnetic resonance imaging (MRI). Schematic illustrating changes between healthy versus fibrotic kidneys and how these pathological alterations are hypothesized to be reflected by changes in quantitative MRI parameters (arterial spin labeling [ASL], longitudinal relaxation time [T1], apparent diffusion coefficient [ADC], and transverse relaxation time [T2*]). The colored boxes indicate the physiological and structural properties that are hypothesized to be captured by the respective quantitative MRI measures. ADC: apparent diffusion coefficient; ASL: arterial spin labeling; MRI: magnetic resonance imaging; T1: longitudinal relaxation time; T2*: effective transverse relaxation time.

### Molecular Biomarkers of Renal Aging, Tubular Integrity, and Fibrosis

Although mpMRI provides spatial information on whole-organ structure and function, urinary, circulating, and tissue biomarkers may provide complementary molecular information on renal aging, tubular integrity, nephron mass, and fibrogenesis. Cellular senescence is increasingly recognized as a contributor to renal aging and chronic tissue remodeling. Senescent cells undergo irreversible growth arrest and may develop a pro-inflammatory and pro-fibrotic secretory phenotype [16,17]. Tissue expression of p16INK4a, a marker of cellular senescence, has been associated with renal aging and adverse transplant outcomes [18,19].

Markers related to tubular health may provide additional insights into functional nephron mass and tubulointerstitial injury. Serum uromodulin has been proposed as a marker of tubular mass and renal parenchymal integrity, whereas urinary epidermal growth factor normalized to urinary creatinine has been associated with kidney function, tubulointerstitial integrity, and graft outcomes [20-23]. Klotho is an antiaging protein expressed in the kidney, and reduced Klotho expression has been linked to renal aging, fibrosis, and kidney injury [24,25]. Together, these biomarkers may help distinguish structural fibrosis from functional, inflammatory, hemodynamic, or aging-related changes. Integrating molecular biomarkers with mpMRI may therefore improve biological interpretation of longitudinal imaging findings.

### Knowledge Gaps and Study Objectives

To date, longitudinal studies combining repeated mpMRI, reference-standard mGFR, serial renal allograft biopsies, and molecular biomarker assessment remain limited. Thus, there is a lack of prospective data validating these imaging parameters against quantitative histological fibrosis. To the best of our knowledge, no study has simultaneously compared imaging and functional trajectories across LKDs, KTRs, and HCs.

The primary objective of the multiparametric renal magnetic resonance imaging (MPRENAL) study is to evaluate the association between time-dependent changes in the corticomedullary T1 difference and progression of histologically determined fibrosis in KTRs, with further MRI measures examined in a fixed order specified in advance of analysis. Secondary objectives are to assess associations between MRI measures and mGFR, evaluate longitudinal divergence of imaging and functional markers across LKD, KTR, and HC cohorts, and explore diagnostic performance for clinically relevant fibrosis thresholds. A composite MRI index derived by principal component analysis (PCA) will be evaluated as an exploratory analysis.

We hypothesize that time-dependent increases in renal fibrosis in KTRs will be associated with a decreasing corticomedullary T1 difference (ΔT1), reflecting progressive loss of corticomedullary differentiation, and with a decreasing corticomedullary ADC difference (ΔADC) and decreasing ASL-derived perfusion. We further hypothesize that MRI measures and biomarkers of renal aging, fibrosis, and nephron mass will be associated with serial mGFR measurements and that longitudinal trajectories of MRI measures and functional markers in KTRs and LKDs will diverge from those in HC. These hypotheses are addressed through a single prespecified primary estimand in KTRs, with the remaining imaging measures being examined in a prespecified fixed order; the analytical hierarchy is described in the statistical analysis plan.

## Methods

### Study Design and Setting

This is a multicenter, prospective, longitudinal observational cohort study including LKDs, KTRs, and HCs. Participants will undergo repeated assessments over a 24-month follow-up period. As illustrated in Figure 2, the study follows a longitudinal trajectory over 24 months for all 3 cohorts. Following the baseline assessment, participants undergo follow-up visits at 3, 12, and 24 months. These visits include repeated mpMRI, mGFR measured using [^99m^Tc]Tc-diethylenetriaminepentaacetic acid (DTPA), and blood and urine biomarker collection to capture the dynamic progression of renal remodeling and functional changes. For the KTR cohort, protocol biopsies are integrated into this timeline to provide histological validation of the imaging markers at these intervals. A detailed timeline of all data collection procedures and visit windows is provided in Table S1 (Multimedia Appendix 1).

**Figure 2.**
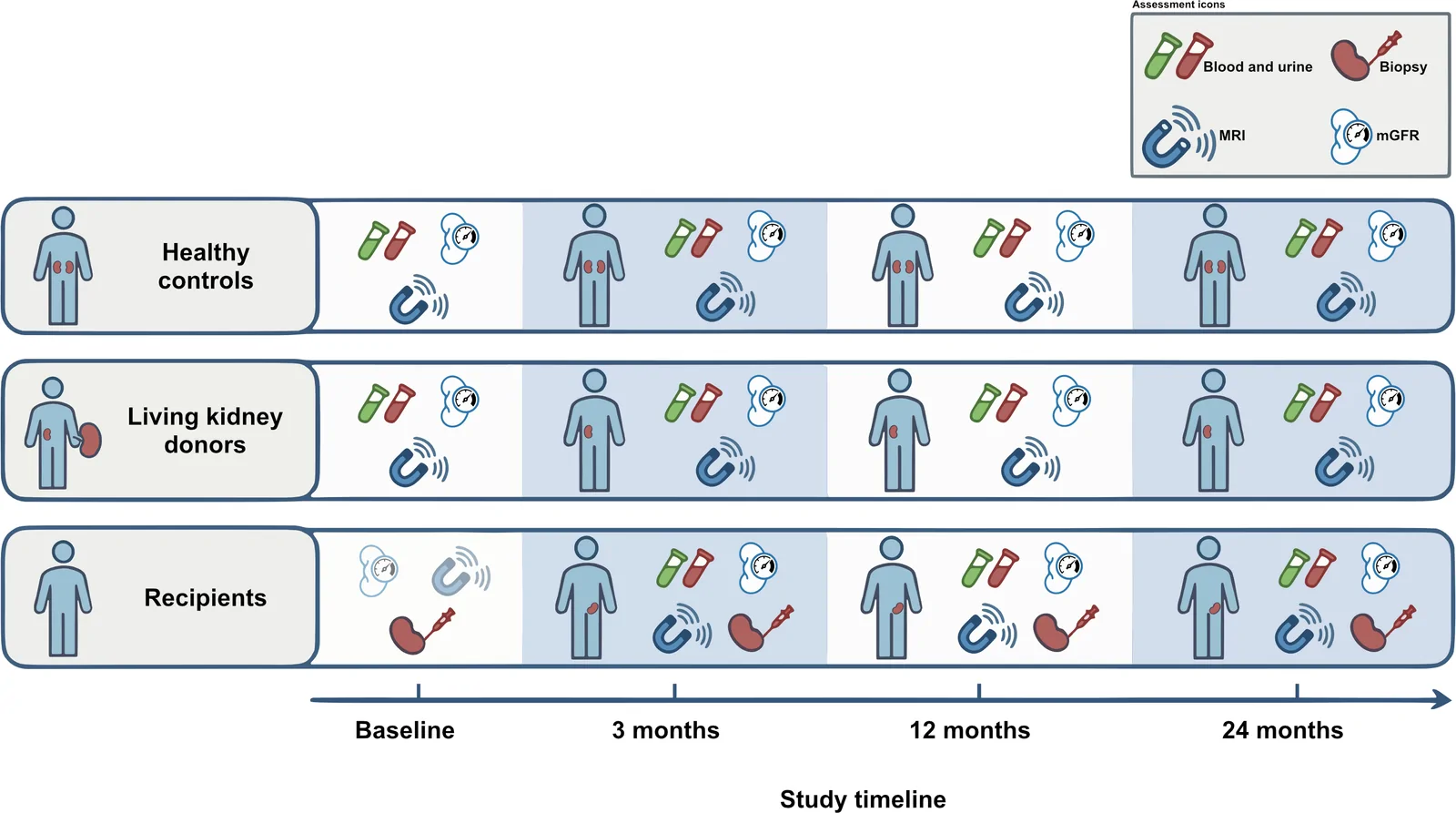
Longitudinal study design for kidney donors, recipients, and healthy controls. Overview of the comprehensive 24-month study protocol, including baseline and follow-up assessments with magnetic resonance imaging (MRI), [99mTc]Tc-diethylenetriaminepentaacetic acid (DTPA), biopsy (recipients only), and biomarker collection at 3, 12, and 24 months. mGFR: measured glomerular filtration rate; MRI: magnetic resonance imaging.

### Reporting Framework

This study protocol is reported in accordance with the STROBE (Strengthening the Reporting of Observational Studies in Epidemiology) statement for observational studies, with adaptations appropriate for a prospective, longitudinal cohort protocol. Items relating to final participant flow, outcome data, and main results will be reported in accordance with STROBE in subsequent results publications. Elements relevant to diagnostic performance analyses will additionally be informed by Standards for Reporting of Diagnostic Accuracy Studies (STARD) recommendations.

### Protocol Amendments and Registry Reconciliation

This protocol is reported after recruitment commenced, and all changes to the protocol and to the trial registry record are therefore documented with dates in Table S3 (Multimedia Appendix 1). Recruitment opened on October 29, 2024. No amendment affecting the eligibility criteria, the outcome measures, the target sample size, the study design, or study procedures has been made since the first participant was enrolled; the registered outcome measures and target enrollment remain unchanged from the initial registration of January 18, 2024. The single change of protocol version recorded by the research ethics committee, from version 2 to version 3 on June 26, 2024, concerned the addition of a funding source and minor changes to research group membership. This change preceded the first enrollment and was accepted by the committee as notification of a minor change rather than as a substantial amendment.

Two categories of change are nevertheless recorded for transparency. First, the statistical analysis plan was developed in full during preparation of this manuscript, after recruitment had commenced; this includes the Monte Carlo power simulation, the corticomedullary difference and ratio variables, the composite MRI index, the diagnostic thresholds, and the reproducibility assessment. The target sample size was fixed at the design stage on feasibility grounds and was not altered by any of this work. Second, several registry entries were corrected where the registered text no longer described the procedure applied in practice, specifically the estimated scan duration, the biopsy needle gauge, and an instruction on fluid intake that was never implemented. These corrections are itemized in Table S3 in Multimedia Appendix 1.

Analytical decisions were made at 3 distinct stages. These stages are distinguished explicitly because the analysis plan was prospectively specified in its entirety. Decisions made prospectively and registered before recruitment opened on October 29, 2024, comprise the study design, the 3 cohorts, the eligibility criteria, the assessment schedule, the target sample size, and the registered outcome measures. None of these decisions have been amended since. Decisions made after recruitment commenced but before any analysis of study data comprise the statistical analysis plan in its present form, including linear mixed modeling, the corticomedullary difference and ratio variables, the composite MRI index, the diagnostic thresholds, and the reproducibility assessment. These were specified between April 2025 and August 2026 and are dated individually in Table S3 in Multimedia Appendix 1. The primary MRI measure was selected in August 2026. At that point, the mpMRI examinations accrued to date had been reviewed for image quality and for the feasibility of corticomedullary segmentation, and the review informed the choice of the corticomedullary T1 difference in preference to the corticomedullary ADC difference. No association between any MRI measure and histological fibrosis had been examined, and no biopsy data had been analyzed, and the selection was therefore made without knowledge of the primary outcome.

### Temporal Definition of Baseline

Baseline definitions differ across cohorts and reflect the biological context of each group. In HCs, baseline mpMRI and mGFR are obtained at the time of the baseline visit and on the same calendar day. In LKDs, baseline mpMRI is obtained at the study baseline visit before donation. Baseline mGFR is based on the clinical predonation mGFR measurement performed as part of the standard donor workup. As part of the same clinical evaluation, renography is performed to estimate split renal function. The total donor mGFR and split renal function are used to estimate the baseline single-kidney GFR of the kidney that is subsequently donated and transplanted. The clinical predonation mGFR and renography may be performed at a variable interval before the baseline mpMRI; this interval will be calculated and reported.

In KTRs, baseline measurements are conceptualized at the level of the kidney rather than the recipient. Specifically, baseline mpMRI and estimated single-kidney GFR are derived from the donor’s pretransplant assessments of the kidney that is subsequently transplanted. Baseline histological fibrosis is obtained from the time-zero biopsy performed at implantation.

Accordingly, baseline measures in KTR reflect the intrinsic preexisting state of the donor kidney rather than a steady-state posttransplant physiological condition. Associations between baseline MRI measures and time-zero histological fibrosis are interpreted accordingly. They describe relationships between donor-derived structural and functional characteristics and underlying tissue composition.

Given the relatively slow progression of interstitial fibrosis, time-zero biopsy findings are considered representative of preexisting tissue remodeling in the donor kidney. However, it is acknowledged that transplantation introduces physiological changes, including ischemia-reperfusion injury, hemodynamic alterations, and exposure to immunosuppressive therapy. These may influence subsequent measurements.

Temporal baseline definitions are provided in Table 1.

**Table 1.** Temporal baseline definitions for mpMRI^a^, mGFR^b^, and fibrosis.

Cohort	Baseline definition	mpMRI values used	Measured GFR^c^ or estimated single-kidney GFR value used	Fibrosis value used
HC^d^	Date of baseline visit	mpMRI from baseline visit	DTPA^e^-GFR from baseline visit	Not applicable
LKD^f^	Date of baseline visit (predonation)	mpMRI from baseline visit	Clinical predonation DTPA-GFR performed as part of standard donor workup; interval to baseline mpMRI recorded	Time-zero biopsy (biopsy of donated kidney performed at transplantation)
KTR^g^	Time of transplant surgery	Predonation mpMRI of the donated kidney (from corresponding donor)	Estimated single-kidney GFR of the donated kidney derived from donor predonation DTPA-GFR and renography	Time-zero biopsy of the implanted kidney

a mpMRI: multiparametric magnetic resonance imaging.

b mGFR: measured glomerular filtration rate.

c GFR: glomerular filtration rate.

d HC: healthy control.

e DTPA: diethylenetriaminepentaacetic acid.

f LKD: living kidney donor.

g KTR: kidney transplant recipient.

### Recruitment and Sites

LKD and KTR participants will be recruited from outpatient clinics at the Departments of Renal Medicine at Aalborg University Hospital and Aarhus University Hospital during the clinical evaluation phase for living donor transplantation. Over a 2-year inclusion period, all eligible LKDs and KTRs will be screened. Enrollment is nondyadic, allowing participation of individuals or donor-recipient pairs. HCs will be recruited via digital platforms, including specialized research recruitment websites and targeted social media announcements. HCs will be recruited during a later phase of the study to allow group matching to the observed age and sex distribution of the enrolled LKD cohort. This strategy is intended to improve comparability between HCs and LKDs and avoid recruiting a demographically mismatched control cohort.

### Eligibility and Attrition

To ensure long-term data integrity and follow-up compliance, the following will not be considered for enrollment: (1) individuals residing abroad unlikely to attend follow-up visits, and (2) individuals with insufficient proficiency in Danish or English to provide informed consent or complete study procedures. Detailed eligibility, including MRI safety and renal function requirements, is provided in Table 2. Recruitment, enrollment, follow-up, biopsy completion, and inclusion in analyses will be tracked using a cohort flow diagram adapted for observational longitudinal studies.

**Table 2.** Specification of inclusion and exclusion criteria.

Cohort	Inclusion criteria	Exclusion criteria
LKD^a^ and KTR^b^	Age ≥18 years Approved for transplantation	Contraindications to MRI^c^ Severe claustrophobia
HC^d^	Age ≥18 years Office BP^e^ <140/90 mm Hg Normal eGFR^f^ (2009 CKD-EPI^g^) Dipstick negative for hematuria and proteinuria Urine albumin-to-creatinine ratio <30 mg/g BMI ≤ 30 kg/m²	Contraindications to MRI Severe claustrophobia Pregnancy Morbidities that would exclude living kidney donation

a LKD: living kidney donor.

b KTR: kidney transplant recipient.

c MRI: magnetic resonance imaging.

d HC: healthy control.

e BP: blood pressure.

f eGFR: estimated glomerular filtration rate.

g CKD-EPI: Chronic Kidney Disease Epidemiology Collaboration.

### Ethical Considerations

This study is conducted in accordance with the Declaration of Helsinki and has received approval from the North Denmark Region Committee on Health Research Ethics (N-20230053) [26]. Written informed consent will be obtained from all participants prior to any study-related procedures. The study complies with the General Data Protection Regulation; all participant data will be pseudonymized and stored securely. The project is registered under the North Denmark Region’s internal record of processing activities (F2023-135).

Potential LKD and KTR candidates will be asked by nurses in the outpatient clinic if they are interested in receiving information regarding this study. Interested candidates will be contacted by the principal investigator (PS) to discuss the study in further detail. Potential participants will receive comprehensive oral and written information regarding the study’s objectives, procedures, and potential risks. Information sessions will be conducted in a quiet, private setting by the principal investigator or a trained member of the research team. To ensure a supportive decision-making environment, participants are encouraged to bring a companion (eg, a family member or friend). In accordance with Danish ethical guidelines, a reflection period of up to 7 days will be offered after the initial information session. Participants will be explicitly informed that consent is voluntary and can be withdrawn at any time without any impact on their current or future clinical treatment.

A separate, secondary informed consent will be sought for the long-term storage of blood and urine samples in a research biobank. The biobank supports future exploratory studies within the scope of the approved protocol. This process will follow the same ethical principles and documentation standards as the primary study consent. Except for transport reimbursement, participants will not receive financial compensation for their participation.

### Data Collection and Procedures

#### Variable Definitions

Definitions, units, and derivation of study variables are provided in Table S2 (Multimedia Appendix 1). This list is not exhaustive but reflects the key variables relevant to the predefined study objectives. Each variable is recorded at the study visits indicated in Table S1 in Multimedia Appendix 1.

#### Blood Pressure

Blood pressure will be measured in accordance with the standardized clinical operating procedures at the participating hospitals using an automated office blood pressure device. After an initial rest period, the device is programmed to automatically perform 6 consecutive measurements at 2‐5-minute intervals using an appropriately sized arm cuff. To minimize the white-coat effect and ensure an unattended measurement, the procedure is conducted in a quiet, private room where the participant remains alone during the automated cycles. Participants are blinded to the results during the procedure. The first measurement is automatically discarded, and the final recorded value is the calculated average of the remaining 5 measurements. In instances where standardized office blood pressure is not available (eg, during inpatient ward stays or urgent clinical visits), blood pressure values obtained via validated hospital-grade electronic sphygmomanometers (office blood pressure) will be recorded. Home blood pressure monitoring data are also acceptable for this study.

#### Multiparametric MRI Acquisition and Analysis

To ensure longitudinal comparability, identical acquisition parameters will be maintained across study sites and all visits. Individual participants will be scanned on the same MRI system throughout the study. Participants will be instructed to refrain from eating for 3 hours prior to each scan; intake of liquids will be permitted. In accordance with PARENCHIMA recommendations, participants attend in a normally hydrated state, and no fixed fluid-loading protocol is applied; the 3-hour interval without food is not specified in those recommendations and was adopted by the investigators to reduce postprandial variation in renal hemodynamics between visits [27].

Imaging will be performed on a 3.0 T GE Signa Premier scanner or a 3.0 T GE Signa 750 (GE Healthcare) using a 32-channel body array coil. Because each participant is scanned on the same system throughout the study, scanner identity is a strictly between-participant factor and is absorbed by the participant-level random intercept in all longitudinal models. Study site is additionally retained as a covariate in adjusted models. The large majority of kidney transplant recipients, in whom the primary analysis is conducted, are examined at a single site, so the primary analysis is effectively single-scanner. The mpMRI protocol has been harmonized with the technical recommendations of the PARENCHIMA COST Action (CA16103) to facilitate future meta-analyses and multicenter comparisons.

The comprehensive protocol (approximate scan time 45 min) includes the following sequences, where detailed acquisition parameters are provided in Table 3:

- Anatomical imaging: T2-weighted single-shot fast spin echo (SSFSE) and T1-weighted gradient echo sequences for morphological assessment and kidney volume segmentation.
- DWI: a respiratory-triggered spin-echo echo-planar imaging (SE-EPI) sequence with 13 b-values (0‐800 s/mm^2^).
- BOLD (transverse relaxation time [T2*]) mapping: a multiple gradient-recalled echo (mGRE) sequence acquiring 12 echoes (echo time [TE] 1=5.0 ms; ΔTE=4.3 ms) to map T2* as a surrogate for renal tissue oxygenation.
- T1 and T2 mapping: a modified Look-Locker inversion recovery (MOLLI) sequence (5-(3)-3 scheme) for T1 mapping and a multiecho fast spin echo sequence for T2 mapping.
- ASL: A 2D pseudocontinuous ASL (pCASL) sequence with SSFSE readout and background suppression to quantify renal blood flow (RBF).
- Phase contrast (PC-MRI): cardiac-gated phase contrast imaging of the renal arteries to quantify bulk blood flow.

**Table 3.** Multiparametric MRI^a^ acquisition parameters.

Sequence	Technique and readout	Key parameters	Note
Anatomical (volumetry)	Breath-hold Coronal-oblique T2^b^-weighted SSFSE^c^	TR^d^ and TE^e^: 1165 ms and 60 ms Flip angle: 90° FOV^f^: 38.4 cm Matrix: 256×256 Resolution: 1.5×1.5×5.0 mm	Used for kidney volume segmentation and morphological assessment.
DWI^g^	Respiratory triggered Coronal-oblique SE-EPI^h^	b-values: 0, 10, 20, 30, 40, 50, 70, 100, 200, 300, 400, 500, 800 s/mm² TR and TE: 5000 and 51 ms FOV: 38.4 cm Matrix: 128×128 Resolution: 3.0×3.0×5.0 mm	Complies with consensus-based technical recommendations for DWI MRI [28].
BOLD^i^ (T2*^j^) mapping	Breath-hold Coronal-oblique mGRE^k^	Echoes: 12 (TE: 5‐52 ms) Echo spacing: 4.3 ms TR: 80 ms Flip angle: 18° FOV: 38.4 cm Matrix: 256×256 Resolution: 1.5×1.5×5.0 mm	Complies with consensus-based technical recommendations for renal BOLD MRI [29]. Uses mGRE with >8 echoes and TE range covering expected renal T2^∗^.
T1^l^ mapping	Cardiac gated, breath-hold Coronal-oblique MOLLI^m^ 5(3)3	TI^n^: 270‐3301 ms Flip angle: 20° FOV: 38.4 cm Matrix: 256×256 Resolution: 1.5×1.5×5.0 mm	Complies with consensus-based technical recommendations for T1 and T2 mapping MRI [27] Uses the standardized MOLLI 5(3)3 scheme for robust breath-hold quantification
T2 mapping	Respiratory triggered Coronal-oblique Multiecho fast spin echo	Echoes: 10 (TE: 10‐104 ms) TR: >1500 ms FOV: 38.4 cm Matrix: 128×128 Resolution: 3.0×3.0×4.5 mm	Complies with consensus-based technical recommendations for T1 and T2 mapping MRI [27]
ASL^o^	Free-breathing Coronal 2D pCASL^p^ (SSFSE readout)	Label duration: 1525 ms Postlabel delay: 1525 ms Labeling distance: 80 mm Background suppression: Yes FOV: 42 cm Matrix: 128×128 Resolution: 3.3×3.3×5.0 mm	Complies with consensus-based technical recommendations for renal ASL MRI [30] Uses recommended postlabel delay (>1500 ms) and background suppression for robustness
Phase contrast	Cardiac gated Breath-hold 2D cine phase contrast	Orientation: Perpendicular renal artery Flip angle: 20° VENC^q^: 100 cm/s Temporal resolution: 40 cardiac phases FOV: 28 cm Matrix: 188×188 Resolution: 1.49×1.49×6 mm	Complies with consensus-based technical recommendations for renal phase-contrast MRI [31]

a MRI: magnetic resonance imaging.

b T2: transverse relaxation time.

c SSFSE: single-shot fast spin echo.

d TR: repetition time.

e TE: echo time.

f FOV: field of view.

g DWI: diffusion-weighted imaging.

h SE-EPI: spin-echo echo-planar imaging.

i BOLD: blood oxygen level-dependent.

j T2*: effective transverse relaxation time.

k mGRE: multiple gradient-recalled echo.

l T1: longitudinal relaxation time.

m MOLLI: modified Look-Locker inversion recovery.

n TI: inversion time.

o ASL: arterial spin labeling.

p pCASL: pseudocontinuous arterial spin labeling.

q VENC: velocity encoding.

#### Image Processing and Analysis

Postprocessing of MRI data will be performed using a combination of commercially available software and Siswin, a locally developed, noncommercial analysis platform created at the MR Research Centre, Department of Clinical Medicine, Aarhus University. To ensure full transparency and reproducibility, Siswin is available to the research community upon request. Use of the Digital Imaging and Communications in Medicine (DICOM) standard will ensure data integrity, portability, and reproducibility. To ensure longitudinal consistency across the 2 MRI systems, all quantitative maps will be generated using identical reconstruction algorithms to ensure that changes in renal tissue are reflective of biological rather than computational shifts. Retrospective motion correction will be applied to DWI and ASL series to correct for respiratory motion between frame acquisitions using (translation and rotation) registration algorithms.

Quantitative parametric maps (ADC, T2*, T1, T2, and RBF) will be generated on a pixel-by-pixel basis:

- T1 maps: signal intensities will be fitted to the following function:

$$S\left(TI\right)=\left|S_{0}\left(1-2e^{-\frac{TI}{T_{1}}}\right)\right|$$

- T2 maps: signal intensities will be fitted to the following function:

$$S\left(TE\right)=S_{0}e^{-\frac{TE}{T_{2}}}$$

- DWI: Monoexponential ADC maps will be calculated using all b-values (0‐800 s/mm^2^):

$$S\left(b\right)=S_{0}e^{-b∙ADC}$$

- BOLD: T2* maps will be generated by mono-exponential fitting of signal intensity decay across the 12 echo times with an echo spacing of 4.3 ms:

$$S\left(TE\right)=S_{0}e^{-\frac{TE}{T_{2}^{*}}}$$

- ASL: RBF will be quantified using the single-compartment model:

$$RBF=\frac{6000∙\lambda ∙{(M}_{control}-M_{label})∙e^{-\frac{PLD}{T_{1,blood}}}}{2∙\alpha ∙T_{1,blood}∙M_{0}∙(1-e^{-\frac{\tau }{T_{1,blood}}})}$$

See Textbox 1 for the definitions of the terms in the renal blood flow equation.

Textbox 1. **Definitions of the terms in the renal blood flow equation.**

λ: blood-tissue partition coefficient

M_control_-M_label_: the perfusion-weighted difference

PLD: postlabeling delay

T_1, blood_: longitudinal relaxation time of arterial blood

α: labeling efficiency

M_0_: proton-density weighted reference image

τ: labeling duration

#### Region of Interest Placement Strategy

##### Overview

Region of interest (ROI) placement will be compliant with the manual ROI methodology recommended by the PARENCHIMA consensus groups to ensure reproducibility and exclusion of artifacts [27-31]. One observer blinded to clinical data and time points will perform the analysis. Due to slight variations in anatomical positioning between sequences, ROIs will be drawn manually on sequence-specific parametric maps or their corresponding reference images (eg, the b=0 s/mm^2^ image for DWI, the proton-density weighted reference image [M0] or T1 map for ASL). The high-resolution coronal-oblique T1-weighted and T2-weighted sequences will serve as the anatomical ground truth to guide all manual segmentations. By using the T1-weighted and T2-weighted scans as references for the cortico-medullary junction and the renal boundaries, the observer can ensure precise tissue classification even on lower-resolution quantitative maps. Figure 3 illustrates the manual segmentation and ROI placement procedure. Cortical and medullary ROIs are drawn manually on the sequence images used to generate the quantitative maps (T1, T2*, ADC, and ASL) and then propagated to the corresponding maps, as indicated by the arrows. Kidney volume is obtained by manually delineating the renal parenchymal border on T2-weighted slices. For renal artery flow quantification, ROIs are defined on phase-contrast MRI magnitude images and subsequently transferred to the corresponding velocity images.

**Figure 3.**
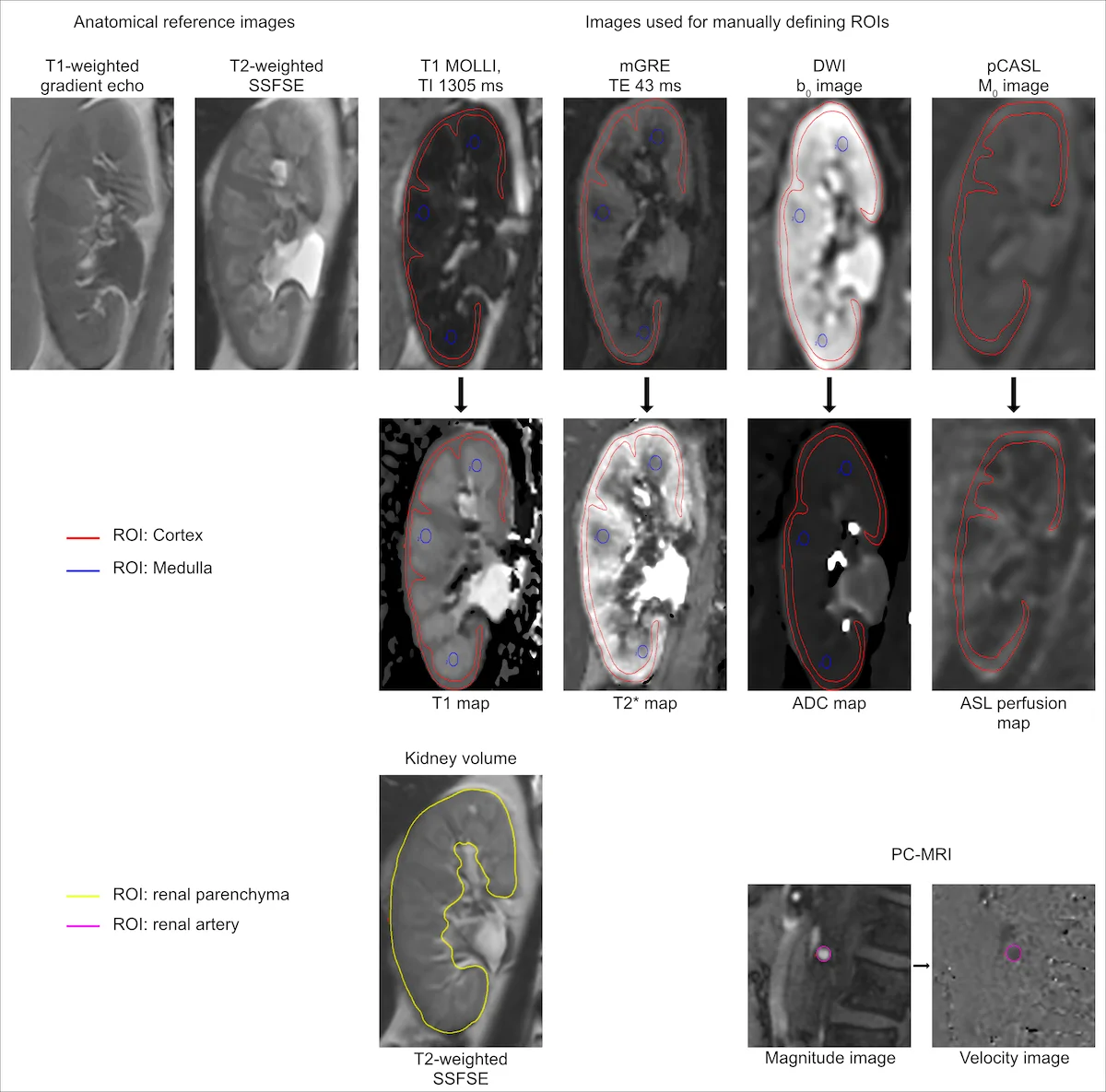
Example of multiparametric renal magnetic resonance imaging (MRI) segmentation in the right kidney of a living donor. ADC: apparent diffusion coefficient; ASL: arterial spin labeling; DWI: diffusion-weighted imaging; mGRE: multiple gradient-recalled echo; MOLLI: modified Look-Locker inversion recovery; pCASL: pseudocontinuous arterial spin labeling; PC-MRI: phase-contrast magnetic resonance imaging; ROI: region of interest; SSFSE: single-shot fast spin echo; TI: inversion time; T2*: effective transverse relaxation time.

##### Kidney Volume

Total kidney volume (TKV) will be determined using manual segmentation on coronal T2-weighted breath-hold images. For each contiguous slice, an ROI encompassing the entire renal parenchyma and all intrarenal cysts will be manually traced. The renal pelvis and hilar structures (vessels and fat) will be strictly excluded to ensure measurement precision. TKV will be calculated by the summation of areas across all slices multiplied by the slice interval.

##### Phase Contrast

Circular ROIs will be manually delineated on each magnitude frame, ensuring complete coverage of the vessel lumen while avoiding partial volume effects at the vessel wall. This frame-by-frame approach eliminates the need for post hoc motion correction. In cases of multiple renal arteries, the total renal blood flow will be calculated as the sum of the flow through the main and accessory arteries.

### Parametric Maps

For the T1-map, T2-map, DWI, BOLD, and ASL sequences, the following procedure will be used:

- Cortical ROIs: for each slice, a continuous freehand cortical stripe ROI will be drawn along the renal cortex, maintaining a margin of 2‐3 mm from the capsule (to avoid partial volume effects with perirenal fat) and 2‐3 mm from the medulla. This will be repeated in at least 3 representative slices.
- Medullary ROIs: for each slice, 3 distinct medullary pyramids will be selected. A circular or elliptical ROI will be placed within the center of each pyramid to strictly avoid the renal columns (cortical tissue) and the renal sinus. Medullary ROIs are not defined for ASL. Areas of cysts, scarring, susceptibility artifacts (eg, bowel gas), and large vessels will be explicitly excluded from all ROIs.
- Reporting: mean values for the cortex and medulla will be averaged across ≥3 slices and weighted by the respective ROI area to provide a single representative whole cortex and whole medulla value per kidney.

### Reproducibility Assessment

To assess intraobserver reproducibility, repeat postprocessing will be performed in a subset of approximately 15 participants. ROIs will be redrawn by the same observer blinded to the original measurements after a washout period. Agreement between repeated measurements will be quantified using intraclass correlation coefficients based on a 2-way mixed effects model with absolute agreement for single measurements. Bland-Altman analysis and relative differences will be used to further describe measurement variability. Agreement will be reported separately for each quantitative measure entering the analytical hierarchy, including ΔT1, cortical T1, ΔADC, and ASL-derived renal blood flow. This will permit direct comparison of the measurement reliability of corticomedullary differences derived from T1 mapping and from DWI in this cohort, which is relevant to the interpretation of any differences in their association with histological fibrosis.

### Allograft Biopsy and Histological Analysis

A surgical allograft needle biopsy will be performed during the transplant surgery in all KTRs. Subsequent voluntary protocol biopsies are planned at 3, 12, and 24 months. Biopsies performed for clinical indications will be collected but will not be pooled indiscriminately with protocol biopsies in the primary analysis. Because indication biopsies are triggered by clinical events and may therefore introduce bias, they will be analyzed separately or included only in predefined sensitivity analyses.

Apart from the surgical allograft biopsy, biopsy specimens are obtained under ultrasound guidance and fixed in phosphate-buffered 4% formaldehyde before paraffin embedding. Biopsies performed at Aarhus University Hospital, which account for the large majority of study biopsies, use an 18G needle; the smaller number performed at Aalborg University use a 16G needle. Needle gauge is therefore determined by study site rather than by participant characteristics or clinical circumstance. Sections will be stained with hematoxylin and eosin, periodic acid-Schiff, and Masson trichrome.

### Quantitative Stereology

Histological evaluation will be performed using an Olympus BX50 light microscope (Olympus Denmark) equipped with a motorized stage and an Olympus DP70 digital camera interfaced with newCAST software (Visiopharm). To ensure objectivity, the extent of interstitial fibrosis will be quantified using a 4 x lens (numerical aperture 0.13) using point counting (approximately 100 test points) across Masson trichrome-stained sections [32]. This stereological approach provides a continuous variable for fibrosis percentage (0%‐100%).

### Immunohistochemistry

Further characterization of renal aging will be achieved through immunohistochemical staining for p16INK4a (a marker of cell-cycle arrest) and Klotho (an antiaging marker). Quantitative stereology (QS) will be used to estimate the number of positive cells. To ensure high-level evidence, all histological and immunohistochemical analyses will be conducted by observers blinded to the MRI data and mGFR.

### [^99m^Tc]Tc-DTPA Clearance (mGFR)

[^99m^Tc]Tc-DTPA clearance is used to measure GFR. [^99m^Tc]Tc-DTPA clearance will be performed according to standard operating procedures (SOP) at the Departments of Nuclear Medicine at the participating hospitals. Gamma counting is performed on plasma samples, and mGFR is determined by the slope-intercept method, using the revised Brøchner-Mortensen correction [33].

The procedure is:

- A peripheral intravenous cannula, with a 3-way stopcock, is inserted;
- A baseline blood sample (time 0) is drawn in a 5.5 mL lithium-heparin tube;
- Subsequently, the calculated DTPA solution is injected;
- Time of injection is recorded;
- The peripheral intravenous cannula is removed;
- If eGFR is >30 mL/min/1.73 m^2^, 4 blood samples are drawn after 180 min, 200 min, 220 min, and 240 min after injection of [^99m^Tc]Tc-DTPA solution;
- If eGFR is ≤30 mL/min/1.73 m^2^ and ≥15 mL/min/1.73 m^2^, an additional blood sample is drawn after 300 min;
- If eGFR is <15 mL/min, a further blood sample is drawn 24 hours after the time of injection; and
- Time of sampling will be recorded.

### mpMRI, mGFR, and Biopsy Temporal Alignment

The study visit schedule and predefined visit windows are provided in Table S1 (Multimedia Appendix 1). Baseline timing definitions for mpMRI, mGFR, renography, and time-zero biopsy are described above. For follow-up assessments in LKD, KTR, and HC, mpMRI and mGFR will be performed on the same calendar day to ensure direct temporal alignment between imaging-derived and functional measures.

Renal allograft biopsy will be performed on a separate calendar day from the mpMRI. When feasible, protocol biopsies will be scheduled within the predefined visit window corresponding to the relevant study visit. However, biopsy timing may be influenced by participant burden, coordination with clinical appointments, and logistical constraints. In addition, biopsy will not be scheduled immediately before mpMRI, as biopsy-related local tissue changes could potentially influence imaging findings.

For all paired mpMRI-biopsy assessments, the exact dates of mpMRI, mGFR, and biopsy will be recorded, and the interval between mpMRI and biopsy will be calculated in days. Primary analyses will use mpMRI-biopsy pairs assigned to the same scheduled study visit. The mpMRI-biopsy interval will be summarized descriptively and considered in sensitivity analyses where feasible, for example by adjusting for interval length or excluding pairs with the longest intervals. Biopsies obtained outside predefined visit windows will be identified, reported, and considered in the interpretation of results.

### Laboratory Measurements

#### Standard Hematological and Biochemical Parameters

Refer to Table 4 for details. These parameters will be analyzed using validated automated assays at the Departments of Clinical Biochemistry at the participating sites (Aarhus University Hospital and Aalborg University Hospital) according to local SOPs. Detailed assay specifications and NPU (nomenclature, properties, and units) codes are available in Danish via the respective laboratory service directories.

eGFR will be calculated using the 2009 Chronic Kidney Disease Epidemiology Collaboration (CKD-EPI) creatinine equation [34]. In accordance with contemporary clinical recommendations to reduce racial bias in glomerular filtration estimation, the race coefficient will be omitted from all calculations. All participants are therefore treated as non-Black. This approach ensures a standardized assessment across the LKD, KTR, and HC cohorts. Blood and urine biomarkers related to tubular integrity and nephron mass—specifically serum uromodulin and urinary epidermal growth factor—will be analyzed batch-wise using commercially available enzyme-linked immunosorbent assays (ELISAs). Batch analysis minimizes interassay variability. Urinary biomarkers will be normalized to urinary creatinine.

**Table 4.** Laboratory measurements.

Category	Parameters
Hematology	Hemoglobin, hematocrit, leukocytes, and platelets
Basic biochemistry	Sodium, potassium, urea, creatinine (eGFR^a^), bicarbonate, glucose, and CRP^b^
Mineral and bone	Ionized calcium, total magnesium, phosphate, alkaline phosphatase, and intact PTH^c^
Urinalysis	Albumin-to-creatinine ratio, glucose, leukocytes, nitrite, and erythrocytes
Research biomarkers	Serum uromodulin and urinary epidermal growth factor

a eGFR: estimated glomerular filtration rate.

b CRP: C-reactive protein.

c PTH: parathyroid hormone.

#### Tacrolimus Monitoring and Quantification

To account for the hemodynamic and vasoconstrictive effects of calcineurin inhibitors (CNIs) on renal perfusion, tacrolimus trough levels will be measured in KTRs immediately prior to MRI examination on the same day. This ensures close temporal alignment between drug exposure and imaging-derived parameters. If a same-day level is unavailable because of logistical or clinical constraints, the clinical trough value most proximal to the date of the MRI will be used.

The vast majority of KTR will be examined at a single study site (Aarhus University Hospital). At that site, tacrolimus concentrations are analyzed using liquid chromatography-tandem mass spectrometry (LC-MS/MS; NPU19912). This consistent assay approach minimizes interassay variability for the primary analyses.

In addition to study-specific measurements, longitudinal tacrolimus concentrations obtained as part of routine clinical care will be available. These measurements may be used in exploratory analyses to better characterize time-varying exposure patterns. It is acknowledged that these values may be derived from different assay platforms across sites and over time. For the KTRs, tacrolimus will be incorporated as a time-varying covariate. When multiple tacrolimus measurements are available between study visits, exploratory analyses will consider summary measures (eg, the mean or area-under-the-curve approximations) to better reflect cumulative exposure.

#### Biobanking and Biomarker Repository

A research biobank will be established at the Department of Renal Medicine, Aalborg University Hospital, to facilitate the standardized collection and long-term storage of biological specimens. Whole blood, serum, plasma, and spot urine samples will be collected at each study visit (baseline, 3, 12, and 24 months) and immediately processed and aliquoted to minimize preanalytical variability. Samples will be stored at –80 °C until batch analysis.

The primary purpose of this repository is to enable the batch-wise quantification of specialized biomarkers—including markers of cellular senescence, tubular integrity, and renal aging—to ensure analytical consistency across the longitudinal study period. Furthermore, this biobank will serve as a resource for future exploratory research into novel injury markers and molecular pathways of renal fibrosis. This integrated approach aims to provide a biological and mechanistic link between systemic molecular changes and the structural alterations observed via mpMRI.

#### Data Management

Source data will be recorded in an electronic case report form using REDCap (Vanderbilt University), a secure, validated clinical data management platform hosted at the North Denmark Region [35,36]. All MRI data will be stored on secure, encrypted institutional servers provided by the North Denmark Region. Imaging data acquired at the Aarhus site are pseudonymized at the source prior to transfer to the central research server at the North Denmark Region. Digital transfer is conducted via FileSender (Danish e-Infrastructure Consortium), a secure, encrypted, open-source web application designed for the transmission of large datasets, ensuring the sender maintains full access control. Alternatively, data may be transferred physically using encrypted USB storage media. Access to the key file linking study IDs to personal identifiers will be restricted to the principal investigator to maintain participant confidentiality. Deidentified aggregate data and the study’s statistical code will be made available upon reasonable request following the publication of primary results, provided such sharing complies with institutional ethics and data privacy regulations.

### Sample Size and Statistical Power

The target sample size of 32 evaluable participants per cohort was fixed at the design stage on feasibility grounds, before recruitment opened. It was based on the annual volume of living-donor transplantation at the 2 participating centers, an inclusion period of 2 years, and an anticipated 20% attrition rate. The sample size was registered on this basis in January 2024 and has not been altered since. For the KTR cohort, up to 40 participants may therefore be recruited to allow for withdrawal, incomplete protocol biopsy participation, incomplete mpMRI data, and loss to follow-up, with the aim of obtaining approximately 32 evaluable KTR with longitudinal mpMRI-biopsy data for the primary analysis.

The simulations described below were performed after recruitment had commenced. They characterize the statistical power of this existing design and were not used to determine it. Because this is an exploratory study using a novel mpMRI protocol, prior data to inform estimates of variance, diagnostic accuracy, and longitudinal change are limited. The power described applies to the primary longitudinal association between ΔT1 and fibrosis in KTRs, while analyses involving LKD, HC, diagnostic performance, biomarkers, and the PCA-derived composite MRI index are secondary or exploratory and are not individually powered.

Based on previous work, we expect that 40%-50% of renal allografts may have significant levels of fibrosis after 2 years [37,38]. However, contemporary fibrosis prevalence may be lower because of improved immunosuppressive regimens. Moderate to large effect sizes for MRI measures, including T1, ADC, and ASL, have been observed between no fibrosis and moderate fibrosis (Banff ci0 vs ci2) [39,40].

Power was characterized by Monte Carlo simulation of a linear mixed effects model (LMM) in the KTR cohort. An initial simulation, based on a composite MRI predictor, was documented in October 2025; following the decision to designate the composite index as exploratory, the simulation was repeated in December 2025 for a single MRI measure, and it is that simulation that is reported here. The simulated model regressed histological fibrosis on time, and the MRI measure entered as a time-varying covariate, with a random intercept for participant, across the 4 planned biopsy time points at 0, 3, 12, and 24 months. Parameters were 32 evaluable KTR with 4 repeated measures, and an intrasubject correlation of 0.6 for fibrosis and 0.7 for the MRI measure, a residual SD of 10 percentage points, and a single fixed-effect coefficient tested at an unadjusted 2-sided α of .05.

Under these assumptions, the existing design provides approximately 84% power to detect a change of 5 percentage points in fibrosis per effect size (*β*=5.0), representing a 5-percentage-point change in fibrosis per 1 SD change in the MRI measure. This corresponds to a standardized association of approximately 0.30, consistent with the association reported between ΔT1 and biopsy-determined fibrosis in kidney allografts [41], and conservative relative to correlations reported in native chronic kidney disease [42]. Power is sensitive to the assumed intrasubject correlation of the MRI measure, which is not established for this protocol. The planned intraobserver reproducibility assessment will provide an initial estimate of the measurement component of this variation. The unadjusted α of .05 is the basis of the multiplicity strategy described below.

The simulation assumes 4 paired mpMRI-biopsy assessments per participant. All participants with at least one valid pair will be included in the primary analysis, as LMMs accommodate unbalanced data under a missing-at-random assumption, and exclusion would introduce selection bias. Participants with fewer assessments contribute proportionally less information. Simulations indicate that the design retains approximately 77% power if 75% of follow-up biopsies are completed and approximately 68% if half are completed.

### Statistical Analysis Plan

#### General Principles

Statistical analyses will be conducted using R version 4.x or later (R Foundation for Statistical Computing). Longitudinal modeling will be implemented using the *tidyverse*, *lme4,* and *lmerTest* packages for LMMs [43-45]. For all models, a 2-sided *P*<.05 will be considered statistically significant. Effect estimates will be reported with 95% CIs throughout.

LMMs will be used to handle longitudinal data, as they are robust to data missing at random. They also account for the inherent correlation between repeated measurements within participants. Model diagnostics, including assessments of normality of residuals and homoscedasticity, will be performed for all LMMs to ensure model fit.

Given the anticipated sample size, model complexity will be guided by data availability, model convergence, and clinical interpretability. Random slopes and interaction terms will be retained only when supported by the data. If models fail to converge or produce unstable estimates, simplified models with random intercepts and reduced covariate sets will be used. The primary analyses will therefore prioritize individual MRI measures and prespecified covariates, while more complex multiparametric models will be considered exploratory.

#### Descriptive Statistics

Baseline characteristics, including demographic, clinical, laboratory data, and imaging measurements, will be summarized for each of the 3 cohorts: LKD, KTR, and HC. Continuous variables will be presented as means with SD or medians with IQRs based on their distribution (assessed by Shapiro-Wilk tests and Q-Q plots). Categorical variables will be reported as frequencies and percentages. Inter-cohort comparisons at baseline will be performed using one-way ANOVA or Kruskal-Wallis tests for continuous data and chi-square or Fisher exact tests for categorical data.

#### Derived Variables

To quantify the loss of corticomedullary differentiation, we will calculate both differences and ratios for T1, T2, T2*, and ADC:

- Corticomedullary difference (CMD): Calculated as Value_medulla_ – Value_cortex_ for T1 and T2 and Value_cortex_ – Value_medulla_ for ADC so that positive values reflect typical anatomy.
- Corticomedullary ratio (CMR): Calculated as Value_cortex_ / Value_medulla_ to provide a dimensionless, internally normalized index of tissue homogeneity.

Both CMD and CMR values will be entered into the PCA to evaluate their contribution to the composite MRI index and will be modeled longitudinally using LMM. Loss of CMD is a recognized feature of progressive renal impairment and of allograft injury, driven by preferential prolongation of cortical T1 toward medullary values [46,47]. Note that the ΔT1 is defined here as medullary minus cortical T1, so that positive values indicate preserved CMD and values are expected to fall as fibrosis progresses. Several published studies define the difference in the opposite direction and accordingly report a positive association with fibrosis; the 2 conventions describe the same relationship with an inverted sign.

#### Primary Analysis: Longitudinal Association of MRI With Histological Fibrosis

##### Primary Estimand

The primary MRI measure is ΔT1, calculated as T1 in the renal medulla minus T1 in the renal cortex. The primary estimand is the association between ΔT1 and interstitial fibrosis, quantified stereologically as the percentage of cortical area in KTRs. It is estimated as the fixed-effect coefficient for ΔT1 entered as a time-varying covariate in an LMM across the 4 protocol biopsy time points at 0, 3, 12, and 24 months and is expressed as the difference in fibrosis in percentage points per 1 SD of ΔT1. The primary measure is therefore specified by contrast (T1 mapping), region (medulla minus cortex), and by time structure (all 4 biopsy time points jointly). We hypothesize an inverse association, as progressive interstitial fibrosis is expected to reduce CMD.

A corticomedullary difference was selected in preference to a single-compartment value for 2 reasons. In a prospective study of 71 patients with biopsy-confirmed renal fibrosis, the ΔT1 correlated with histopathological fibrosis score and was an independent predictor of fibrosis on multivariable analysis, whereas cortical T1 alone was not [42]. In kidney allografts, ΔT1 has shown a significant association with biopsy-determined fibrosis, whereas absolute T1 values have not [41,48]. In addition, an internally normalized measure is less sensitive than an absolute value to systematic differences between MRI systems. This is relevant in the present multicenter design.

T1 mapping was selected in preference to DWI as the source of the primary corticomedullary difference. Published comparisons in kidney allografts have reported a stronger association with interstitial fibrosis for ΔADC than for ΔT1 [48], but that comparison used a purpose-developed segmented diffusion-weighted acquisition, which is not used here. In the present cohort, respiratory motion and limited corticomedullary contrast on diffusion-weighted images have made reliable medullary ROI placement more difficult than on T1 maps. ΔADC is retained within the analytical hierarchy, and the reproducibility of both measures will be reported.

The primary model is fibrosis ~ time+ΔT1+age+sex+study site, with a random intercept for each participant. This specification matches the model used in the power simulation. The MRI×time interaction term and random slopes for participants are informative but were not included in the power calculation and are therefore specified as secondary analyses; they will be fitted where the data support convergence and reported as such. Sequential covariate adjustment as described below is likewise secondary.

Defining the estimand across all 4 time points rather than at a single visit is deliberate. The power calculation derives its efficiency from 4 repeated measures with an intrasubject correlation of 0.6 for fibrosis. An analysis restricted to one single time point would not correspond to the design for which the study was powered.

##### Multiplicity

To control the family-wise error rate at 2-sided *α*=.05 without reducing power for the primary estimand, 4 MRI measures will be examined in a fixed sequence, specified in August 2026 before analysis of study data: (1) ΔT1 (primary); (2) cortical T1; (3) ΔADC, calculated as cortex minus medulla; and (4) cortical renal blood flow derived from ASL. Testing proceeds to the next measure in the sequence only if the preceding test reaches *P*<.05. Once a test does not reach this threshold, that measure and all subsequent measures in the sequence are reported descriptively with effect estimates and 95% CIs and are not interpreted as confirmatory. A fixed-sequence procedure controls the family-wise error rate irrespective of the correlation between measures and because the first test is performed at the full alpha, it does not reduce the power of the primary analysis.

The ordering reflects prior evidence, measurement reliability in this cohort, and interpretability. Cortical T1 is placed second because although it is a component of the primary measure and the 2 are therefore correlated, it is the most widely reported single quantitative measure in renal mpMRI and a result expressed in cortical T1 is directly comparable with the existing literature. We note that absolute cortical T1 has not been consistently associated with interstitial fibrosis in all allograft cohorts [48], and its position in the hierarchy reflects comparability rather than an expectation of superior performance.

All remaining MRI measures, including T2 and T2* mapping, cortical and medullary ADC, CMR, kidney volume, and phase-contrast renal artery blood flow, lie outside this hierarchy. These measures are exploratory. Analysis of the composite MRI index, diagnostic performance, circulating biomarkers, and the LKD and HC cohorts are likewise exploratory throughout.

##### Within-Person and Between-Person Associations

As a secondary analysis, specified before any outcome analysis, ΔT1 will be decomposed into a participant-level mean, representing the between-person association, and the deviation of each observation from that mean, representing the within-person association. Both terms will be entered simultaneously in the LMM, and both coefficients will be reported with 95% CIs. This distinguishes whether participants with a lower average ΔT1 have more fibrosis from whether within-participant change in ΔT1 tracks within-participant change in fibrosis. The decomposition is specified as secondary rather than primary because the study was powered for the pooled coefficient; simulation under the original assumptions indicates that the separated components are individually less well powered, and results will therefore be interpreted on effect estimates and CIs rather than on statistical significance.

### Secondary and Exploratory Analyses

#### Development of a Composite MRI Index

To address the high dimensionality and potential collinearity of mpMRI data (eg, correlations between T1, T2, T2*, ADC, and RBF), PCA will be used as a data reduction technique. PCA will be applied to standardized MRI variables across all participants and time points to explore shared patterns of variation in the imaging data. The MRI measures entering PCA will include the predefined quantitative MRI measures with sufficient data completeness and technical quality, including T1, T2, T2*, ADC, ASL-derived renal blood flow where applicable, kidney volume, and derived corticomedullary difference and ratio variables. The final PCA variable set may be restricted to a core set of technically robust variables if the number of candidate variables is high relative to the available sample size.

The proportion of variance explained by the first principal component will be reported. If the first component explains an insufficient proportion of total variance or lacks clinically interpretable loadings, additional components, or alternative dimensionality-reduction approaches will be considered. Participants or visits with missing MRI measures required for PCA will be handled using predefined missing-data procedures. Complete-case PCA will be used as the primary approach if missingness is limited; sensitivity analyses using imputation or restricted variable sets may be performed depending on the pattern and extent of missingness. Given the data-driven nature of PCA, the composite MRI index will be considered exploratory. It is intended to summarize multidimensional imaging information rather than serve as a predefined primary predictor.

To assess the robustness of the PCA-derived components, internal validation will be performed using bootstrap resampling (≥500 iterations) at the participant level. Stability of component loadings and variability of derived indices across resampled datasets will be evaluated. The composite MRI index will be evaluated in parallel analyses to assess whether a multiparametric approach improves associations with fibrosis and mGFR compared to individual MRI measures. Given the exploratory nature of secondary, diagnostic, biomarker, and PCA-derived analyses, results will be interpreted with emphasis on effect estimates and CIs rather than statistical significance alone.

#### Additional Secondary Analyses

The following additional secondary analyses are planned:

- Group divergence: longitudinal trajectories of MRI measures will be compared across the 3 cohorts (LKD, KTR, and HC) using LMMs with a cohort×time interaction term.
- Associations with mGFR: the longitudinal association between mGFR and MRI measures will be assessed using LMMs, with mGFR as the dependent variable.
- Biomarker analyses: similarly, the associations between markers of renal aging and nephron mass and changes in both mGFR and histological fibrosis will be modeled. We will specifically test the hypothesis that an increase in p16ink4a precedes a measurable increase in histological fibrosis.
- Diagnostic performance: receiver operating characteristic analyses will be performed to assess the ability of MRI measures and the composite index to detect clinically relevant fibrosis thresholds (>10% and >25%) at 24 months. Area under the curve will be compared using DeLong test. The fibrosis thresholds of >10% and >25% were selected to evaluate diagnostic performance across clinically relevant degrees of fibrosis, representing mild-to-moderate and more advanced fibrosis. These threshold-based analyses are exploratory and will complement the primary analysis using fibrosis as a continuous variable.

#### Handling of Missing Data

LMMs allow inclusion of participants with incomplete follow-up under a missing-at-random assumption. However, missing biopsy data may not be random, as participants declining protocol biopsies may differ systematically from participants completing all biopsies. Participants with complete and incomplete biopsy follow-up will therefore be compared with respect to baseline demographics, clinical characteristics, kidney function, MRI measures, and relevant transplant-related variables.

All participants with at least one valid mpMRI-biopsy pair will be included in the primary longitudinal analyses. Sensitivity analyses will be performed where feasible to assess the influence of incomplete biopsy follow-up. The potential for informative missingness will be explicitly considered when interpreting associations between MRI measures and histological fibrosis. Sensitivity analyses will also consider the interval between mpMRI and biopsy for paired assessments.

#### Covariate Adjustment Strategy

To evaluate whether MRI measures provide independent prognostic information, multivariable LMMs will be used with sequential adjustment:

- Model 1: MRI measure+time.
- Model 2: Model 1+age+sex+study site (Aalborg vs Aarhus).
- Model 3: Model 2+eGFR+u-albumin/creatinine.

For KTR, tacrolimus exposure is additionally entered as a time-varying covariate in the adjusted models. Measurement, assay method, and handling of repeated clinical values are described in the Methods. Where multiple measurements are available between visits, summary measures of cumulative exposure will be considered in exploratory analyses.

#### Bias and Confounding

Potential sources of bias include selection bias related to participation in protocol biopsies and residual confounding from unmeasured LKD, KTR, and perioperative factors. In KTR, temporal differences between donor-derived pretransplant baseline measurements and posttransplant follow-up assessments may introduce bias. These factors will be addressed through covariate adjustment where feasible and explicitly considered in the interpretation of results. For baseline analyses involving donor mGFR and renography together with baseline mpMRI, the interval between clinical examinations (mGFR and renography) and baseline mpMRI will be summarized and considered in sensitivity analyses where feasible.

## Results

The study was funded in October 2023. Recruitment for the MPRENAL study commenced on October 29, 2024. As of March 2026, a total of 42 participants had been enrolled, including 18 LKDs, 22 KTRs, and 2 HCs. Several participants have completed their initial 3-month and 12-month follow-up examinations. Recruitment of HCs continues as planned, and data collection and follow-up are ongoing. No analyses of the primary or secondary outcomes have been performed. Recruitment is anticipated to close in February 2027, with the final 24-month study visits completed by March 2029. Data analysis is planned for 2029, and the primary results are expected to be published in 2030.

## Discussion

### Strengths

This study is designed to address the need for noninvasive biomarkers of renal fibrosis. A primary strength of this protocol is the use of stereological point-counting on Masson trichrome-stained sections as the reference standard for fibrosis. Unlike the semiquantitative Banff scoring system [49], stereology provides a continuous, objective measure of tubulointerstitial fibrosis, offering a more precise value for MRI validation.

Furthermore, the integration of [^99m^Tc]Tc-DTPA clearance ensures that functional changes are captured by a reference-standard mGFR rather than eGFR. This distinction is important because eGFR can be unreliable in the early posttransplant period [50,51]. By performing serial measurements at baseline and at 3, 12, and 24 months, this study will characterize the temporal evolution of renal injury in both LKDs and KTRs, providing a rare insight into the natural history of the solitary and transplanted kidneys.

If validated, the multiparametric MRI approach evaluated in this study may contribute to future noninvasive assessment of renal fibrosis. A noninvasive, reliable marker of fibrosis could allow for more frequent monitoring of graft health, potentially reducing the reliance on protocol biopsies. It could also enable earlier adjustment of immunosuppressive therapy before injury becomes irreversible.

The integration of a longitudinal biobank allows for a multimodal approach to renal diagnostics. This combines spatial information from mpMRI with the molecular insights from blood and urine biomarkers. The combined use of imaging and biomarker data may enhance the characterization and prediction of renal structural and functional changes.

### Limitations

A key limitation of this study is the spatial mismatch between the focal needle biopsy samples and the cortical and medullary ROIs assessed by mpMRI. A biopsy typically represents less than 1% of the total kidney volume; therefore, the patchy nature of renal fibrosis may lead to discrepancies between the localized histopathology and the whole-organ imaging parameters. Although stereology maximizes the accuracy of the tissue analyzed, it cannot fully mitigate the inherent sampling bias of the biopsy-based assessment.

Additionally, manual segmentation of MRI data for volume estimation and ROI placement introduces the potential for observer bias. To mitigate this, all postprocessing procedures will be standardized and performed by blinded observers. This is intended to ensure that time-dependent shifts in parameters like T1 and ADC reflect biological change rather than observer or computational variability.

An additional limitation relates to temporal alignment of baseline assessments in KTRs, as described in the Methods. Functional and imaging baselines are derived from the donor before transplantation, whereas the histological baseline is derived from the time-zero biopsy. Early posttransplant changes, including ischemia-reperfusion injury, hemodynamic shifts, surgical factors, and immunosuppressive exposure, may influence subsequent measurements. Associations involving baseline should therefore be interpreted with caution. Although tacrolimus levels are measured contemporaneously with MRI assessments in most cases, variability in exposure between visits and differences in assay methods for routine clinical measurements may introduce residual confounding.

Another limitation is that renal allograft biopsy will not be performed on the same calendar day as mpMRI and mGFR. Although protocol biopsies are planned within predefined visit windows whenever feasible, participant burden, coordination with clinical appointments, and logistical constraints may result in variable mpMRI-biopsy intervals, and some biopsies may occur outside the planned visit window. Exact dates will be recorded, intervals will be reported, and sensitivity analyses will be performed where feasible. Nevertheless, temporal separation between mpMRI and biopsy may introduce residual measurement misalignment.

Renal T1 is sensitive to interstitial edema as well as to fibrosis, and reduced ΔT1 has been linked to both. The specificity of T1-based measures for fibrosis specifically is therefore limited, and this is a recognized constraint of renal relaxometry [47]. The concurrent inclusion of circulating and histological biomarkers is intended to assist interpretation.

Participant preparation regarding hydration follows PARENCHIMA consensus recommendations by instructing participants to attend in a normally hydrated state rather than by administering a fixed fluid load [27]. Consequently, physiological hydration status at the time of scanning is not strictly controlled. Between-visit variation in hydration may contribute to variability in T1, ADC, and perfusion measures. This is mitigated in the longitudinal analyses by the reliance on within-participant comparison but cannot be excluded as a source of measurement noise.

The biopsy needle gauge differs between the 2 study sites, which may influence the quantity of cortical tissue available for stereological assessment. Because the gauge is determined entirely by the site, this source of variation is absorbed by the study site covariate. It cannot, however, be separated from other site-related differences. These methodological limitations should be considered when interpreting associations among imaging biomarkers, histological findings, and functional outcomes in the present study.

## Supplementary material

10.2196/97152 Multimedia Appendix 1 This file contains Table S1 (study schema and data collection timeline), Table S2 (variable definitions), and Table S3 (dated protocol amendments and registry updates).

## Abbreviations

3 T: 3 tesla
ADC: apparent diffusion coefficient
ASL: arterial spin labeling
BOLD: blood oxygen level-dependent
CKD-EPI: Chronic Kidney Disease Epidemiology Collaboration
CMD: corticomedullary difference
CMR: corticomedullary ratio
CNI: calcineurin inhibitors
DICOM: Digital Imaging and Communications in Medicine
DTPA: diethylenetriaminepentaacetic acid
DWI: diffusion-weighted imaging
eGFR: estimated glomerular filtration rate
ELISA: enzyme-linked immunosorbent assay
HC: healthy control
KTR: kidney transplant recipient
LC: liquid chromatography
LKD: living kidney donor
LMM: linear mixed effects model
mGFR: measured glomerular filtration rate
mGRE: multiple gradient-recalled echo
MOLLI: modified Look-Locker inversion recovery
mpMRI: multiparametric magnetic resonance imaging
MPRENAL: multiparametric renal magnetic resonance imaging
MS: mass spectrometry
PC-MRI: phase contrast magnetic resonance imaging
PCA: principal component analysis
pCASL: pseudocontinuous arterial spin labeling
QS: quantitative stereology
RBF: renal blood flow
ROI: region of interest
SE-EPI: spin-echo echo-planar imaging
SOP: standard operating procedure
SSFSE: single-shot fast spin echo
STARD: Standards for Reporting of Diagnostic Accuracy Studies
STROBE: Strengthening the Reporting of Observational Studies in Epidemiology
T2*: effective transverse relaxation time
TE: echo time
TKV: total kidney volume
ΔADC: corticomedullary apparent diffusion coefficient difference
ΔT1: corticomedullary inversion time difference
