# Supplementary material for: Multiparametric Magnetic Resonance Imaging in a Prospective Cohort of Living Kidney Donors, Recipients, and Healthy Controls and Correlations With Markers of Renal Function, Fibrosis, and Aging (MPRENAL): Protocol for a Longitudinal Observational Study
Source: JMIR Res Protoc. 2026 Oct 28;15:e97152. doi: 10.2196/97152 (PMC13717433; doi:10.2196/97152)
Supplement: Supplementary file 1 [file resprot-v15-e97152-s001.docx]

# Multimedia Appendix 1: Supplementary Tables

**List of Tables**

[Table S1 Study schema and data collection timeline.	2](#_Toc238050058)

[Table S2 Variable definitions.	3](#_Toc238050059)

[Table S3 Dated protocol amendments and registry updates.	4](#_Toc238050060)

| Visit | Enrolment | | | Visit 1 | | | Visit 2 | | | Visit 3 | | | Visit 4 | | |
| --- | --- | --- | --- | --- | --- | --- | --- | --- | --- | --- | --- | --- | --- | --- | --- |
| Month |  | | | **0** | | | **3** | | | **12** | | | **24** | | |
| Visit windows |  | | |  | | | +/- 2 weeks | | | +/- 4 weeks | | | +/- 4 weeks | | |
| Cohort | **LKD^1^** | **KTR^2^** | **HC^3^** | **LKD** | **KTR** | **HC** | **LKD** | **KTR** | **HC** | **LKD** | **KTR** | **HC** | **LKD** | **KTR** | **HC** |
| Informed consent | x | x | x |  |  |  |  |  |  |  |  |  |  |  |  |
| In- and exclusion criteria | x | x | x |  |  |  |  |  |  |  |  |  |  |  |  |
| Demography | x | x | x |  |  |  |  |  |  |  |  |  |  |  |  |
| Medical history | x | x | x |  |  |  |  |  |  |  |  |  |  |  |  |
| Current medications | x | x | x | x | x | x | x | x | x | x | x | x | x | x | x |
| Vital signs (height^4^, weight, blood pressure, heart rate) | x | x | x | x | x | x | x | x | x | x | x | x | x | x | x |
| Record relevant data electronic patient record | x | x | x | x | x | x | x | x | x | x | x | x | x | x | x |
| Endpoints |  |  |  |  |  |  |  |  |  |  |  |  |  |  |  |
| Multiparametric MRI |  |  |  | x | x^8^ | x | x | x | x | x | x | x | x | x | x |
| Renal allograft biopsy |  |  |  |  | x |  |  | x |  |  | x |  |  | x |  |
| Measured GFR |  |  |  | x | x^9^ | x | x | x | x | x | x | x | x | x | x |
| Blood samples^5^ |  |  |  | x |  | x | x | x | x | x | x | x | x | x | x |
| Blood samples^6^ |  |  | x |  |  |  |  |  |  |  |  |  |  |  |  |
| Urine samples^7^ |  |  | x | x |  | x | x | x | x | x | x | x | x | x | x |
| Blood & urine for Biobank |  |  |  | x |  | x | x | x | x | x | x | x | x | x | x |
|  | | | | | | | | | | | | | | | |
| 1 Living Kidney Donor | | | | | | | | | | | | | | | |
| 2 Kidney Transplant Recipient | | | | | | | | | | | | | | | |
| 3 Healthy Control | | | | | | | | | | | | | | | |
| 4 Height only at Visit 1 | | | | | | | | | | | | | | | |
| 5 Haemoglobin, haematocrit, leukocytes, platelets, CRP, creatinine, eGFR, urea, sodium, potassium, total phosphate, bicarbonate, ionized calcium, total magnesium, intact PTH, glucose and b-tacrolimus (KTR) | | | | | | | | | | | | | | | |
| 6 Estimated Glomerular Filtration Rate | | | | | | | | | | | | | | | |
| 7 Urine dipstick, u-albumin/creatinine ratio | | | | | | | | | | | | | | | |
| 8 Pretransplant multiparametric MRI ~ donated kidney | | | | | | | | | | | | | | | |
| 9 Pretransplant measured GFR ~ donated kidney | | | | | | | | | | | | | | | |

Table S1 Study schema and data collection timeline.

Schedule of visits, study cohorts, and the specific data collection and procedures (e.g., multiparametric MRI, measured GFR, biopsies) to be performed at each time point

| Category | Variable | Definition/Method | Units/Type |
| --- | --- | --- | --- |
| Primary Outcome | Fibrosis | Percentage of tubulointerstitial area affected by interstitial fibrosis, quantified via stereological point-counting on Masson-trichrome sections. | Percentage (%) |
| Secondary Outcomes | mGFR | Measured Glomerular Filtration Rate determined by [^99m^Tc]Tc-DTPA plasma clearance. | mL/min |
|  | T1 Mapping | Longitudinal relaxation time; surrogate for interstitial oedema and fibrosis. Values reported for cortex and medulla. | Milliseconds (ms) |
|  | T2 Mapping | Transverse relaxation time; sensitive to water content of tissue. Values reported for cortex and medulla. | Milliseconds (ms) |
|  | T2* Mapping | Transverse relaxation time; sensitive to local magnetic field inhomogeneities and used as a surrogate for renal tissue oxygenation. Values reported for cortex and medulla. | Milliseconds (ms) |
|  | ADC | Apparent Diffusion Coefficient; reflects water molecular diffusion hindered by fibrosis. Values reported for cortex and medulla. | mm²/s |
|  | ASL | Renal microvascular perfusion measured using Arterial Spin Labelling. | mL/100 g/min |
|  | Corticomedullary Difference (T1, T2, T2*, ADC) | Measures corticomedullary dedifferentiation. Calculated as Value_medulla_ – Value_cortex_ or Value_cortex_ – Value_medulla_ ensuring positive values reflect typical anatomy. | Milliseconds  (ms) |
|  | Corticomedullary Ratio (T1, T2, T2*, ADC) | Internally normalized index of tissue homogeneity. Calculated as Value_cortex_ / Value_medulla._ | Dimensionless |
|  | Phase Contrast | Total arterial blood flow measured via Phase Contrast MRI in the main renal arteries; used to quantify bulk delivery of blood to the kidneys. | mL/min |
|  | Kidney Volume | Volume of renal parenchyma. Measured by manual segmentation of coronal T2-weighted breath-hold images. | mL |
| Assay to be determined | P16INK4a | Marker of cellular senescence in biopsy tissue. Immunohistochemistry. | % area tissue |
| Assay to be determined | Klotho | Marker of renal ageing, with decreasing levels associated with fibrogenesis. Immunohistochemistry. | % area tissue |
| Assay to be determined | u-Epidermal Growth Factor/u-creatinine | Urinary epidermal growth factor normalized to urinary creatinine. Marker of functional nephron mass. | pg/mg or ng/mg |
| Assay to be determined | Serum Uromodulin | Serum marker of functional tubular mass. | ng/mL |
| Assay to be determined | Clusterin | Sensitive early marker of tubular injury. Measurable in urine and localizable in tissue by immunohistochemistry | ng/mL (urine) / % area (tissue) |

Table S2 Variable definitions.

| Date | Source | Change | Relative to recruitment |
| --- | --- | --- | --- |
| 2023-09-29 | Ethics | Informed consent form, biobank sub-study, version 1 | Before |
| 2024-01-04 | Ethics | Study protocol version 2; participant information version 2 (donors, recipients, healthy controls); biobank consent version 2 | Before |
| 2024-01-08 | Registry | Initial registration submitted (NCT06210555) | Before |
| 2024-01-18 | Registry | Record first posted. Outcome measures and target enrolment as registered here and unchanged since | Before |
| 2024-02-06 | Ethics | Final approval, N-20230053, Committee on Health Research Ethics for the North Denmark Region. Approval valid to 1 January 2030 | Before |
| 2024-02-07 | Ethics | Recruitment material version 3 | Before |
| 2024-06-26 | Ethics | Study protocol version 3; participant information version 3. Addition of funding source and minor changes to research group membership | Before |
| 2024-06-27 | Ethics | Committee acknowledged the above as notification of a minor change; no supplementary protocol required | Before |
| 2024-10-08 | Registry | Recruitment status set to recruiting; ethics oversight details, cohort descriptions and site contacts added | Before (21 days) |
| 2024-10-29 | — | **First participant enrolled** | — |
| 2025-01-27 | Registry | Actual study start date recorded; site investigator roles corrected. Administrative only | After |
| 2025-07-23 | Registry | Editorial revision of the study description; keywords added. Instruction that participants drink 1 L of water removed, as it was never implemented and no participant underwent fluid loading | After |
| 2025-10-21 | Analysis | Statistical analysis plan revised and specified in full, including corticomedullary difference and ratio variables, the composite MRI index (exploratory), initial power simulation based on a composite MRI predictor and diagnostic threshold of >10%; documented in the 11-month PhD study plan submitted to the Doctoral School, Aalborg University. | After |
| 2025-12 | Analysis | Monte Carlo simulation repeated for a single MRI measure rather than the composite index, to characterise the power of the fixed sample size for the primary longitudinal analysis. The calculation presented in the present manuscript. Sample size, outcomes, eligibility and recruitment target unchanged | After |
| 2026-04-04 | Analysis | Diagnostic threshold of >25% added alongside the previously specified >10% threshold, to evaluate diagnostic performance across a more advanced degree of interstitial fibrosis. First documented in the original manuscript submitted to JMIR Research Protocols on this date | After |
| 2026-07 | Analysis | Introduced in response to peer review: intraobserver reproducibility assessment; separate handling of indication biopsies; recording of the mpMRI–biopsy interval; bootstrap validation of composite index loadings | After |
| 2026-08 | Analysis | Primary MRI measure and multiplicity hierarchy specified: corticomedullary T1 difference as primary, with a prespecified fixed-sequence order for three further measures. Specified on prior evidence and on image quality assessed in accrued scans, blind to histological outcomes | After |
| 2026-08-19 | Registry | Record updated (posted 20 August 2026): scan duration corrected to 45 minutes; biopsy needle gauge specified by site; power calculation description replaced; analysis plan summarised; anticipated completion dates revised to 31 December 2029; record verification renewed | After |
| 2026-08-25 (Posted) | Registry | Anticipated primary completion date corrected to 1 March 2029, corresponding to the final 24-month study visit for the last participant anticipated to be enrolled. The anticipated study completion date remains 31 December 2029, reflecting completion of data analysis and reporting | After |

Table S3 Dated protocol amendments and registry updates.

Ethics approvals were issued by Den Videnskabsetiske Komité for Region Nordjylland (N-20230053). Registry entries refer to ClinicalTrials.gov record NCT06210555; the complete version history is publicly available at https://clinicaltrials.gov/study/NCT06210555?tab=history.
